# 1-Methyl-4-[1-(1-phenyl­ethyl­idene)-hydrazin-2-yl­idene]-3,4-dihydro-1*H*-2λ^6^,1-benzothia­zine-2,2-dione

**DOI:** 10.1107/S1600536812025743

**Published:** 2012-06-13

**Authors:** Muhammad Shafiq, Islam Ullah Khan, Muhammad Zia-ur-Rehman, Tariq Mahmood, Muhammad Ashfaq, Saeed Ahmad

**Affiliations:** a Department of Chemistry, Government College University, Faisalabad 38040, Pakistan; b Materials Chemistry Laboratory, Department of Chemistry, GC University, Lahore 54000, Pakistan; c Applied Chemistry Research Center, PCSIR Laboratories Complex, Ferozpur Road, Lahore 54600, Pakistan; d Department of Chemistry, COMSATS institute of Information Technology, Abbottabad, Pakistan; e Department of Chemistry, University of Gujrat, Gujrat 50781, Pakistan; f Department of Chemistry, Gomal University, Dera Ismail Khan, K.P.K, Pakistan

## Abstract

In the title compound, C_17_H_17_N_3_O_2_S, the phenyl ring is oriented at dihedral angles of 8.5 (2) and 1.17 (14)°, respectively, to the C=N—N plane and the fused aromatic ring. The thia­zine ring adopts an envelope conformation with the S atom at the flap. In the crystal, a weak C—H⋯O inter­action connects the mol­ecules, forming a helical chain along the *a* axis.

## Related literature

For the synthesis, see: Shafiq *et al.* (2011 ▶). For related structures, see: Shafiq *et al.* (2011*a* ▶,*b* ▶, 2012 ▶).
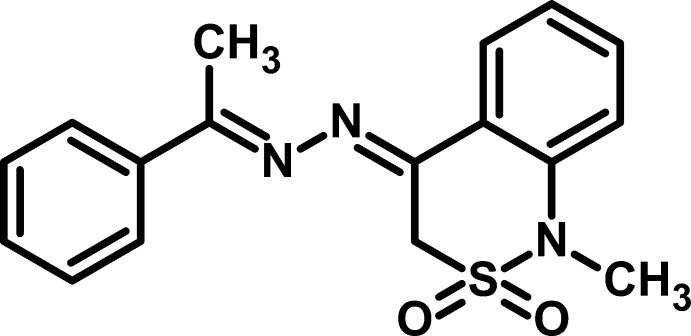


## Experimental

### 

#### Crystal data

C_17_H_17_N_3_O_2_S
*M* *_r_* = 327.40
Orthorhombic, 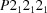

*a* = 6.6678 (2) Å
*b* = 12.0783 (6) Å
*c* = 20.0529 (8) Å
*V* = 1614.97 (11) Å^3^
*Z* = 4
Mo *K*α radiation
μ = 0.21 mm^−1^
*T* = 296 K
0.38 × 0.11 × 0.07 mm

#### Data collection

Bruker Kappa APEXII CCD diffractometer
Absorption correction: multi-scan (*SADABS*; Bruker, 2007 ▶) *T*_min_ = 0.923, *T*_max_ = 0.985
9111 measured reflections
3830 independent reflections
2819 reflections with *I* > 2σ(*I*)
*R*_int_ = 0.030

#### Refinement

*R*[*F* ^2^ > 2σ(*F* ^2^)] = 0.045
*wR*(*F* ^2^) = 0.108
*S* = 0.97
3828 reflections
210 parameters
H-atom parameters constrained
Δρ_max_ = 0.21 e Å^−3^
Δρ_min_ = −0.27 e Å^−3^
Absolute structure: Flack (1983 ▶), 1577 Friedel pairs
Flack parameter: −0.06 (9)

### 

Data collection: *APEX2* (Bruker, 2007 ▶); cell refinement: *SAINT* (Bruker, 2007 ▶); data reduction: *SAINT*; program(s) used to solve structure: *SHELXS97* (Sheldrick, 2008 ▶); program(s) used to refine structure: *SHELXL97* (Sheldrick, 2008 ▶); molecular graphics: *PLATON* (Spek, 2009 ▶); software used to prepare material for publication: *WinGX* (Farrugia, 1999 ▶) and *PLATON*.

## Supplementary Material

Crystal structure: contains datablock(s) I, New_Global_Publ_Block. DOI: 10.1107/S1600536812025743/is5153sup1.cif

Structure factors: contains datablock(s) I. DOI: 10.1107/S1600536812025743/is5153Isup2.hkl

Supplementary material file. DOI: 10.1107/S1600536812025743/is5153Isup3.cml

Additional supplementary materials:  crystallographic information; 3D view; checkCIF report

## Figures and Tables

**Table 1:** Hydrogen-bond geometry (Å, °)

*D*—H⋯*A*	*D*—H	H⋯*A*	*D*⋯*A*	*D*—H⋯*A*
C8—H8*B*⋯O1^i^	0.97	2.56	3.420 (3)	148

Symmetry code: (i) 
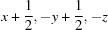
.

**Fig. 1.**
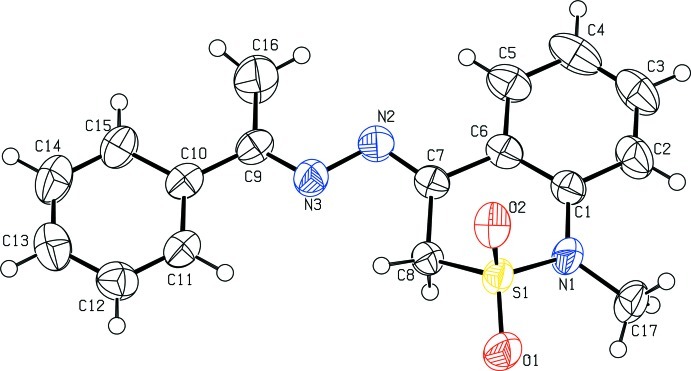
The molecular structure of the title compound, with 50% displacement ellipsoids for non-H atoms.

**Fig. 2.**
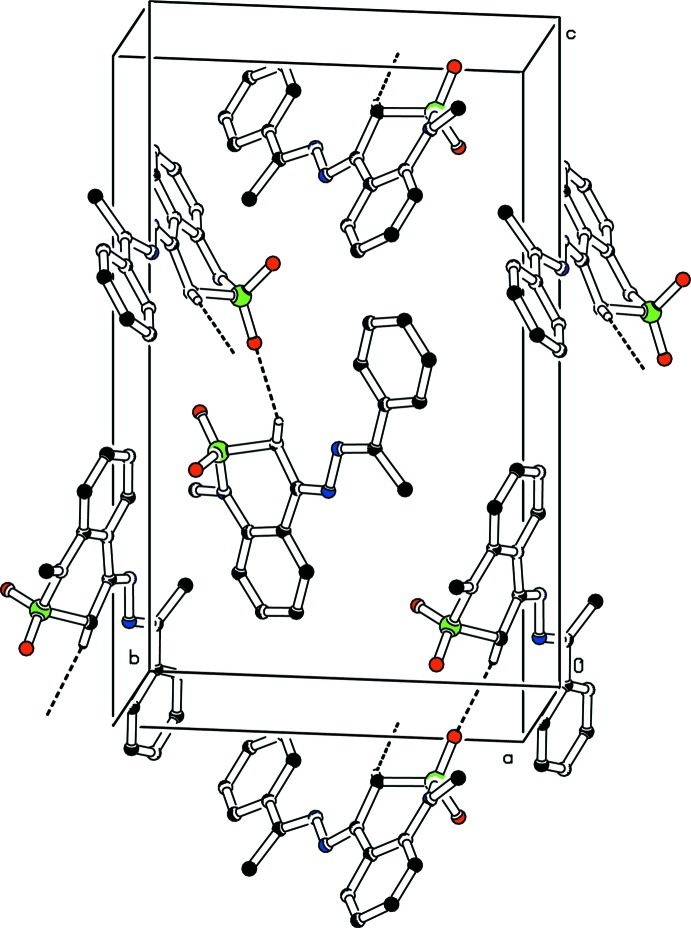
A packing diagram, showing C—H···O hydrogen bonds (dashed lines).

## supplementary crystallographic information

### Comment

The present structure is analogue to 1-ethyl-4-[1-(1-phenylethylidene)-hydrazin-2-ylidene]-3,4-dihydro-1*H*- 2λ^6^,1-benzothiazine-2,2-dione(II) (Shafiq *et al.*, 2012) and related to 4-hydrazinylidene-1-methyl-3*H*-2λ^6^,1-benzothiazine-2,2-dione (III) (Shafiq, Khan *et al.*, 2011*a*) and 6-bromo-1-methyl-4-[2- (4-methylbenzylidene)hydrazinylidene]-3*H*-2λ^6^, 1-benzothiazine-2,2-dione (IV) (Shafiq, Khan *et al.*, 2011*b*). The structure of molecule looks planer as the plane generated from atoms (C1–C7/C9–C15/N1) showes an r.m.s. deviation of 0.0365 Å, while atoms S1 and C16 show maximum deviations of 0.702 (2) and -0.256 (4) Å, respectively. The fused aromatic ring and the mean plane of the thiazine ring are oriented at a dihedral angle of 9.34 (14)° and thiazine ring adopted sofa shape with an r.m.s. deviation of 0.233 (2)°. Comparison of dihedral angles between the phenyl and fused aromatic rings in I and II [*i.e.* 1.17 (14) and 79.33 (2)°, respectively] also explain the planarity of title compound. Similarly dihedral angles between the phenyl and thiazine rings in I and II are 9.34 (2) and 69.74 (6)°, respectively. An intermolecular hydrogen bonding interaction of C—H···O type connects the molecule along the *a* axis and generates a chain structure (Table 1 and Fig. 2). In the group *R*_2_C=N—N=C(CH_3_)Ar, the configurations around the two double bonds are *Z* and *E*, respectively.

### Experimental

In the synthesis of title compound, 4-hydrazinylidene-1- methyl-3*H*-2λ^6^,1-benzothiazine-2,2-dione (Shafiq, Khan *et al.*, 2011*a*) was subjected to react with acetophenone according to literature procedure (Shafiq, Zia-ur-Rehman *et al.*, 2011). The product obtained was then recrystallized in ethylacetate under slow evaporation to obtain single crystals suitable for X-ray diffraction.

### Refinement

All H atoms were positioned with idealized geometry with C—H = 0.93 Å for aromatic, 0.96 Å for methyl group and 0.97 Å for methylene and were refined using a riding model, with *U*_iso_(H) = 1.2*U*_eq_(C) for aromatic and methylene, and *U*_iso_(H) = 1.5*U*_eq_(C) for methyl carbon atoms. Two reflections (0 1 1) and (0 0 2) were omitted in the final refinement.

### Crystal data

**Table:**

C_17_H_17_N_3_O_2_S	*F*(000) = 688
*M**_r_* = 327.40	*D*_x_ = 1.347 Mg m^−^^3^
Orthorhombic, *P*2_1_2_1_2_1_	Mo *K*α radiation, λ = 0.71073 Å
Hall symbol: P 2ac 2ab	Cell parameters from 2415 reflections
*a* = 6.6678 (2) Å	θ = 2.6–21.1°
*b* = 12.0783 (6) Å	µ = 0.21 mm^−^^1^
*c* = 20.0529 (8) Å	*T* = 296 K
*V* = 1614.97 (11) Å^3^	Needle, colorless
*Z* = 4	0.38 × 0.11 × 0.07 mm

### Data collection

**Table:**

Bruker Kappa APEXII CCD diffractometer	3830 independent reflections
Radiation source: fine-focus sealed tube	2819 reflections with *I* > 2σ(*I*)
Graphite monochromator	*R*_int_ = 0.030
φ and ω scans	θ_max_ = 28.3°, θ_min_ = 2.0°
Absorption correction: multi-scan (*SADABS*; Bruker, 2007)	*h* = −8→6
*T*_min_ = 0.923, *T*_max_ = 0.985	*k* = −16→13
9111 measured reflections	*l* = −26→18

### Refinement

**Table:**

Refinement on *F*^2^	Secondary atom site location: difference Fourier map
Least-squares matrix: full	Hydrogen site location: inferred from neighbouring sites
*R*[*F*^2^ > 2σ(*F*^2^)] = 0.045	H-atom parameters constrained
*wR*(*F*^2^) = 0.108	*w* = 1/[σ^2^(*F*_o_^2^) + (0.0554*P*)^2^ + 0.0282*P*] where *P* = (*F*_o_^2^ + 2*F*_c_^2^)/3
*S* = 0.97	(Δ/σ)_max_ = 0.007
3828 reflections	Δρ_max_ = 0.21 e Å^−^^3^
210 parameters	Δρ_min_ = −0.27 e Å^−^^3^
0 restraints	Absolute structure: Flack (1983), 1577 Friedel pairs
Primary atom site location: structure-invariant direct methods	Flack parameter: −0.06 (9)

### Special details

**Table:**

Geometry. All e.s.d.'s (except the e.s.d. in the dihedral angle between two l.s. planes) are estimated using the full covariance matrix. The cell e.s.d.'s are taken into account individually in the estimation of e.s.d.'s in distances, angles and torsion angles; correlations between e.s.d.'s in cell parameters are only used when they are defined by crystal symmetry. An approximate (isotropic) treatment of cell e.s.d.'s is used for estimating e.s.d.'s involving l.s. planes.
Refinement. Refinement of *F*^2^ against ALL reflections. The weighted *R*-factor *wR* and goodness of fit *S* are based on *F*^2^, conventional *R*-factors *R* are based on *F*, with *F* set to zero for negative *F*^2^. The threshold expression of *F*^2^ > σ(*F*^2^) is used only for calculating *R*-factors(gt) *etc*. and is not relevant to the choice of reflections for refinement. *R*-factors based on *F*^2^ are statistically about twice as large as those based on *F*, and *R*- factors based on ALL data will be even larger.

### Fractional atomic coordinates and isotropic or equivalent isotropic displacement parameters (Å^2^)

**Table:**

	*x*	*y*	*z*	*U*_iso_*/*U*_eq_	
C1	−0.0436 (3)	0.1646 (2)	0.19872 (12)	0.0434 (6)	
C2	−0.1882 (4)	0.1755 (3)	0.24813 (14)	0.0607 (8)	
H2	−0.2979	0.2216	0.2412	0.073*	
C3	−0.1708 (5)	0.1190 (3)	0.30686 (14)	0.0689 (9)	
H3	−0.2704	0.1259	0.3390	0.083*	
C4	−0.0087 (5)	0.0522 (3)	0.31910 (14)	0.0703 (9)	
H4	0.0036	0.0154	0.3596	0.084*	
C5	0.1365 (5)	0.0404 (2)	0.27030 (12)	0.0592 (7)	
H5	0.2473	−0.0044	0.2785	0.071*	
C6	0.1196 (4)	0.09462 (19)	0.20875 (11)	0.0425 (5)	
C7	0.2735 (3)	0.07398 (19)	0.15723 (11)	0.0401 (5)	
C8	0.2426 (3)	0.12341 (18)	0.08901 (11)	0.0394 (5)	
H8A	0.1559	0.0756	0.0629	0.047*	
H8B	0.3705	0.1291	0.0662	0.047*	
C9	0.6894 (3)	−0.07684 (19)	0.12801 (12)	0.0448 (6)	
C10	0.8400 (3)	−0.08935 (19)	0.07412 (12)	0.0446 (6)	
C11	0.8205 (4)	−0.0311 (2)	0.01409 (12)	0.0485 (6)	
H11	0.7109	0.0153	0.0078	0.058*	
C12	0.9605 (4)	−0.0414 (2)	−0.03551 (13)	0.0573 (7)	
H12	0.9457	−0.0014	−0.0748	0.069*	
C13	1.1223 (4)	−0.1103 (3)	−0.02763 (15)	0.0666 (8)	
H13	1.2158	−0.1178	−0.0617	0.080*	
C14	1.1456 (4)	−0.1680 (3)	0.03080 (16)	0.0723 (8)	
H14	1.2560	−0.2141	0.0365	0.087*	
C15	1.0061 (4)	−0.1578 (2)	0.08111 (15)	0.0571 (7)	
H15	1.0234	−0.1975	0.1204	0.068*	
C16	0.6984 (5)	−0.1505 (3)	0.18763 (15)	0.0859 (11)	
H16A	0.6132	−0.1214	0.2220	0.129*	
H16B	0.8339	−0.1541	0.2037	0.129*	
H16C	0.6538	−0.2234	0.1756	0.129*	
C17	−0.2573 (3)	0.2746 (2)	0.12031 (13)	0.0601 (8)	
H17A	−0.3647	0.2230	0.1275	0.090*	
H17B	−0.2542	0.2957	0.0742	0.090*	
H17C	−0.2782	0.3391	0.1474	0.090*	
N1	−0.0679 (3)	0.22309 (19)	0.13822 (10)	0.0543 (6)	
N2	0.4234 (3)	0.01227 (18)	0.17172 (10)	0.0500 (5)	
N3	0.5596 (3)	−0.00040 (18)	0.11964 (10)	0.0508 (5)	
O1	0.0707 (2)	0.29407 (17)	0.03230 (9)	0.0663 (6)	
O2	0.2661 (2)	0.32168 (14)	0.13476 (9)	0.0559 (5)	
S1	0.13459 (7)	0.25435 (5)	0.09576 (3)	0.04299 (16)	

### Atomic displacement parameters (Å^2^)

**Table:**

	*U*^11^	*U*^22^	*U*^33^	*U*^12^	*U*^13^	*U*^23^
C1	0.0355 (12)	0.0530 (15)	0.0417 (13)	−0.0110 (11)	0.0034 (10)	−0.0029 (11)
C2	0.0496 (15)	0.073 (2)	0.0592 (17)	−0.0102 (14)	0.0168 (12)	−0.0038 (15)
C3	0.072 (2)	0.080 (2)	0.0548 (18)	−0.0237 (17)	0.0264 (15)	−0.0051 (16)
C4	0.103 (2)	0.063 (2)	0.0452 (16)	−0.0274 (18)	0.0170 (17)	0.0015 (14)
C5	0.0776 (17)	0.0510 (16)	0.0489 (16)	−0.0075 (15)	0.0031 (15)	0.0062 (12)
C6	0.0466 (13)	0.0416 (13)	0.0392 (12)	−0.0097 (11)	0.0021 (11)	−0.0003 (10)
C7	0.0427 (12)	0.0382 (12)	0.0395 (13)	−0.0067 (10)	0.0019 (10)	0.0023 (10)
C8	0.0385 (12)	0.0417 (12)	0.0380 (12)	−0.0005 (9)	0.0027 (10)	0.0002 (11)
C9	0.0459 (13)	0.0354 (13)	0.0531 (14)	−0.0007 (10)	−0.0081 (11)	0.0037 (11)
C10	0.0443 (13)	0.0360 (12)	0.0536 (14)	−0.0027 (10)	−0.0086 (11)	−0.0010 (10)
C11	0.0454 (13)	0.0447 (15)	0.0555 (16)	0.0030 (11)	−0.0077 (11)	0.0006 (12)
C12	0.0661 (17)	0.0553 (17)	0.0506 (16)	−0.0018 (14)	−0.0047 (14)	−0.0050 (13)
C13	0.0637 (17)	0.067 (2)	0.0694 (19)	0.0017 (16)	0.0120 (16)	−0.0144 (17)
C14	0.0625 (17)	0.064 (2)	0.090 (2)	0.0226 (16)	0.0003 (18)	−0.0040 (17)
C15	0.0568 (15)	0.0448 (15)	0.0695 (18)	0.0119 (12)	−0.0036 (14)	0.0044 (13)
C16	0.096 (2)	0.080 (2)	0.081 (2)	0.0307 (19)	0.0198 (18)	0.0376 (19)
C17	0.0329 (12)	0.075 (2)	0.0724 (18)	0.0058 (12)	−0.0040 (11)	−0.0046 (15)
N1	0.0322 (9)	0.0787 (17)	0.0521 (12)	0.0092 (10)	0.0048 (8)	0.0099 (12)
N2	0.0512 (12)	0.0504 (13)	0.0486 (12)	0.0059 (10)	0.0022 (9)	0.0078 (10)
N3	0.0485 (11)	0.0534 (13)	0.0506 (13)	0.0095 (10)	0.0044 (9)	0.0074 (10)
O1	0.0577 (10)	0.0845 (15)	0.0567 (11)	0.0191 (10)	0.0038 (9)	0.0243 (10)
O2	0.0494 (9)	0.0457 (10)	0.0726 (12)	−0.0038 (8)	0.0088 (9)	−0.0036 (9)
S1	0.0344 (3)	0.0471 (3)	0.0475 (3)	0.0042 (3)	0.0046 (2)	0.0087 (3)

### Geometric parameters (Å, º)

**Table:**

C1—C2	1.389 (3)	C10—C11	1.400 (3)
C1—C6	1.392 (3)	C11—C12	1.369 (3)
C1—N1	1.413 (3)	C11—H11	0.9300
C2—C3	1.366 (4)	C12—C13	1.371 (4)
C2—H2	0.9300	C12—H12	0.9300
C3—C4	1.371 (4)	C13—C14	1.372 (4)
C3—H3	0.9300	C13—H13	0.9300
C4—C5	1.384 (4)	C14—C15	1.378 (4)
C4—H4	0.9300	C14—H14	0.9300
C5—C6	1.402 (3)	C15—H15	0.9300
C5—H5	0.9300	C16—H16A	0.9600
C6—C7	1.477 (3)	C16—H16B	0.9600
C7—N2	1.280 (3)	C16—H16C	0.9600
C7—C8	1.507 (3)	C17—N1	1.453 (3)
C8—S1	1.743 (2)	C17—H17A	0.9600
C8—H8A	0.9700	C17—H17B	0.9600
C8—H8B	0.9700	C17—H17C	0.9600
C9—N3	1.277 (3)	N1—S1	1.6402 (19)
C9—C10	1.483 (3)	N2—N3	1.392 (3)
C9—C16	1.491 (3)	O1—S1	1.4251 (18)
C10—C15	1.389 (3)	O2—S1	1.4288 (18)
			
C2—C1—C6	119.8 (2)	C11—C12—C13	120.5 (3)
C2—C1—N1	119.0 (2)	C11—C12—H12	119.8
C6—C1—N1	121.14 (19)	C13—C12—H12	119.8
C3—C2—C1	120.6 (3)	C14—C13—C12	119.7 (3)
C3—C2—H2	119.7	C14—C13—H13	120.1
C1—C2—H2	119.7	C12—C13—H13	120.1
C2—C3—C4	121.0 (3)	C13—C14—C15	120.2 (3)
C2—C3—H3	119.5	C13—C14—H14	119.9
C4—C3—H3	119.5	C15—C14—H14	119.9
C3—C4—C5	119.1 (3)	C14—C15—C10	121.1 (3)
C3—C4—H4	120.5	C14—C15—H15	119.4
C5—C4—H4	120.5	C10—C15—H15	119.4
C4—C5—C6	121.2 (3)	C9—C16—H16A	109.5
C4—C5—H5	119.4	C9—C16—H16B	109.5
C6—C5—H5	119.4	H16A—C16—H16B	109.5
C1—C6—C5	118.3 (2)	C9—C16—H16C	109.5
C1—C6—C7	123.0 (2)	H16A—C16—H16C	109.5
C5—C6—C7	118.8 (2)	H16B—C16—H16C	109.5
N2—C7—C6	118.8 (2)	N1—C17—H17A	109.5
N2—C7—C8	122.9 (2)	N1—C17—H17B	109.5
C6—C7—C8	118.23 (19)	H17A—C17—H17B	109.5
C7—C8—S1	110.21 (16)	N1—C17—H17C	109.5
C7—C8—H8A	109.6	H17A—C17—H17C	109.5
S1—C8—H8A	109.6	H17B—C17—H17C	109.5
C7—C8—H8B	109.6	C1—N1—C17	121.73 (19)
S1—C8—H8B	109.6	C1—N1—S1	117.79 (15)
H8A—C8—H8B	108.1	C17—N1—S1	119.25 (17)
N3—C9—C10	115.9 (2)	C7—N2—N3	113.76 (19)
N3—C9—C16	124.3 (2)	C9—N3—N2	115.0 (2)
C10—C9—C16	119.7 (2)	O1—S1—O2	118.72 (12)
C15—C10—C11	117.4 (2)	O1—S1—N1	107.15 (10)
C15—C10—C9	121.8 (2)	O2—S1—N1	110.61 (11)
C11—C10—C9	120.8 (2)	O1—S1—C8	111.07 (12)
C12—C11—C10	121.0 (2)	O2—S1—C8	107.78 (10)
C12—C11—H11	119.5	N1—S1—C8	99.87 (11)
C10—C11—H11	119.5		
			
C6—C1—C2—C3	0.7 (4)	C11—C12—C13—C14	−1.0 (4)
N1—C1—C2—C3	179.1 (2)	C12—C13—C14—C15	0.7 (5)
C1—C2—C3—C4	1.4 (5)	C13—C14—C15—C10	−0.2 (5)
C2—C3—C4—C5	−1.5 (5)	C11—C10—C15—C14	−0.1 (4)
C3—C4—C5—C6	−0.5 (4)	C9—C10—C15—C14	−179.5 (2)
C2—C1—C6—C5	−2.6 (3)	C2—C1—N1—C17	−13.5 (4)
N1—C1—C6—C5	179.0 (2)	C6—C1—N1—C17	164.9 (2)
C2—C1—C6—C7	176.5 (2)	C2—C1—N1—S1	153.8 (2)
N1—C1—C6—C7	−1.8 (3)	C6—C1—N1—S1	−27.8 (3)
C4—C5—C6—C1	2.5 (4)	C6—C7—N2—N3	−179.38 (19)
C4—C5—C6—C7	−176.7 (2)	C8—C7—N2—N3	2.9 (3)
C1—C6—C7—N2	176.9 (2)	C10—C9—N3—N2	−178.1 (2)
C5—C6—C7—N2	−3.9 (3)	C16—C9—N3—N2	1.0 (4)
C1—C6—C7—C8	−5.3 (3)	C7—N2—N3—C9	−167.5 (2)
C5—C6—C7—C8	173.8 (2)	C1—N1—S1—O1	169.67 (19)
N2—C7—C8—S1	−145.02 (19)	C17—N1—S1—O1	−22.8 (2)
C6—C7—C8—S1	37.3 (2)	C1—N1—S1—O2	−59.5 (2)
N3—C9—C10—C15	170.5 (2)	C17—N1—S1—O2	108.0 (2)
C16—C9—C10—C15	−8.7 (4)	C1—N1—S1—C8	53.8 (2)
N3—C9—C10—C11	−8.9 (3)	C17—N1—S1—C8	−138.6 (2)
C16—C9—C10—C11	172.0 (3)	C7—C8—S1—O1	−169.41 (15)
C15—C10—C11—C12	−0.2 (4)	C7—C8—S1—O2	58.95 (17)
C9—C10—C11—C12	179.2 (2)	C7—C8—S1—N1	−56.59 (17)
C10—C11—C12—C13	0.7 (4)		

### Hydrogen-bond geometry (Å, º)

**Table:**

*D*—H···*A*	*D*—H	H···*A*	*D*···*A*	*D*—H···*A*
C8—H8*B*···O1^i^	0.97	2.56	3.420 (3)	148

Symmetry code: (i) *x*+1/2, −*y*+1/2, −*z*.

## References

[bb1] Bruker (2007). *SADABS*, *APEX2* and *SAINT* Bruker AXS Inc., Madison, Wisconsin, USA.

[bb2] Farrugia, L. J. (1999). *J. Appl. Cryst.* **32**, 837–838.

[bb3] Flack, H. D. (1983). *Acta Cryst.* A**39**, 876–881.

[bb4] Shafiq, M., Khan, I. U., Arshad, M. N., Bukhari, I. H. & Ejaz, (2012). *Acta Cryst.* E**68**, o1927. 10.1107/S1600536812022982 PMC3379482 22719680

[bb5] Shafiq, M., Khan, I. U., Zia-ur-Rehman, M., Arshad, M. N. & Asiri, A. M. (2011*a*). *Acta Cryst.* E**67**, o2038. 10.1107/S1600536811027577 PMC3213487 22091066

[bb6] Shafiq, M., Khan, I. U., Zia-ur-Rehman, M., Arshad, M. N. & Asiri, A. M. (2011*b*). *Acta Cryst.* E**67**, o2092. 10.1107/S1600536811028406 PMC3213534 22091111

[bb7] Shafiq, M., Zia-ur-Rehman, M., Khan, I. U., Arshad, M. N. & Khan, S. A. (2011). *J. Chil. Chem. Soc.* **56**, 527–531.

[bb8] Sheldrick, G. M. (2008). *Acta Cryst.* A**64**, 112–122. 10.1107/S0108767307043930 18156677

[bb9] Spek, A. L. (2009). *Acta Cryst.* D**65**, 148–155. 10.1107/S090744490804362X PMC2631630 19171970

